# Minimal disease activity and impact of disease in psoriatic arthritis: a Spanish cross-sectional multicenter study

**DOI:** 10.1186/s13075-017-1277-1

**Published:** 2017-03-29

**Authors:** Rubén Queiro, Juan D. Cañete, Carlos Montilla, Miguel Abad, María Montoro, Susana Gómez, Ana Cábez, on behalf of the MAAPS study group, J. C. Torre Alonso, J. A. Román-Ivorra, J. Sanz, J. Salvatierra, J. Calvo-Alén, A. Sellas, F. J. Rodriguez, A. Bermúdez, M. Romero, M. Riesco, J. C. Cobeta, F. Medina, A. Aragón, M. L. García, A. Urruticoechea, C. M. González, E. Judez, B. González, P. Fernández, L. Pantoja, R. Morlá

**Affiliations:** 1 Department of Rheumatology, HU, Central de Asturias, Av. Roma, s/n, 33011 Oviedo, Asturias Spain; 2 Department of Rheumatology, HU, Clinic and IDIBAPS, C/Villarroel, 170-08036 Barcelona, Spain; 3 Department of Rheumatology, HU, Parque Natural de las Batuecas 17, Cabrerizos, Salamanca Cp 37193 Spain; 4 Department of Rheumatology HU, Virgen del Puerto, Paraje Valcorchero s/n, 10600 Plasencia, Spain; 5 Medical Department, Pfizer, Avenida de Europa, 20 B, 28108 Alcobendas, Madrid Spain

**Keywords:** Minimal disease activity, Psoriatic arthritis, PsAID, Therapeutic objective

## Abstract

**Background:**

Patients with psoriatic arthritis (PsA) experience functional impairment and reduced quality of life, and thus patient global assessment in PsA is explained mainly by the physical, but also by the psychological, aspect of the disease. To assess the prevalence of minimal disease activity (MDA) in Spanish patients with PsA, we examined their characteristics and the association between MDA and the impact of the disease as assessed by the PsA Impact of Disease (PsAID) questionnaire.

**Methods:**

A cross-sectional multicenter study was carried out in patients who fulfilled the Classification for Psoriatic Arthritis (CASPAR) criteria with at least 1 year of disease duration, and who were treated with biological or conventional synthetic (cs) disease-modifying anti-rheumatic drugs (DMARDs) according to routine clinical practice in Spain. Patients were considered in MDA if they met at least 5/7 of the MDA criteria. The association between MDA and the recently developed PsAID questionnaire was also recorded.

**Results:**

Of 227 patients included, 133 (58.6%) were in the MDA state (52% with antitumor necrosis factor (anti-TNF)α monotherapy, 24% with csDMARD monotherapy, and 24% with anti-TNFα in combination with csDMARD). Using multivariate logistic regression analysis, male gender (odds ratio (OR) 2.74, *p* = 0.001), a sedentary lifestyle (OR 3.13, *p* = 0.002), familial history of PsA (OR 0.38, *p* = 0.036), C-reactive protein (CRP) level (OR 0.92, *p* = 0.010), and use of corticoids (OR 0.33, *p* = 0.007) were considered features related to MDA. MDA patients had a significantly lower impact of the disease according to PsAID (mean total score (SD): MDA 3.3 (3.1) vs. non-MDA 7.1 (5.2); *p* < 0.001).

**Conclusions:**

Nearly 60% of Spanish PsA patients achieve MDA in routine clinical practice. MDA remains one of the most useful therapeutic targets for PsA since patients who reached this state also had a significantly lower impact of disease according to PsAID.

## Background

Psoriatic arthritis (PsA) is a chronic inflammatory musculoskeletal disease that is usually seronegative for rheumatoid factor, associated with psoriasis, and with a prevalence of 0.02–0.42% in the general population and 13.8–30% among patients with psoriasis [1, 2]. PsA is a heterogeneous condition with articular and extra-articular manifestations including a combination of peripheral arthritis, axial disease, enthesitis, dactylitis, and skin and nail disease.

The target of therapy for PsA is to reach a state of remission or, at least, minimal disease activity (MDA). For patients with PsA, the heterogeneity among disease manifestations as well as the need to validate outcome measures makes the definition of remission challenging [3]. Clinical remission requires achieving disease quiescence in all disease domains [3]. In 2010, Coates et al. [4] developed a composite outcome measure as a target of treatment for patients with PsA that encompasses most of the disease domains. These criteria for MDA were validated using interventional trial data [5]. Recent studies have found that MDA could be a reliable target for antitumor necrosis factor alpha (anti-TNF) therapy [6–8].

PsA has a high impact on the lives of patients [9, 10]. Patients with PsA experience significant disability and reduced quality of life, resulting from emotional distress and functional impairment associated with psoriatic skin lesions, as well as arthritis-related joint pain [9, 10]. A prominent benefit of treatment with anti-TNF in PsA patients has been an improvement in patient-reported outcomes (PROs), including health-related quality of life [11]. Recently Gossec et al. have developed and validated the PsA Impact of Disease (PsAID) questionnaire which can be used to calculate a score reflecting the impact of PsA on the lives of patients [12].

Measures that assess outcomes in rheumatic diseases should not only capture the major domains of the disease, but also capture the vision of the patient for improvement in their overall health. The aim of this study was to determine the MDA rate in patients with PsA, to describe their characteristics, and to evaluate the association between MDA and the impact of the disease as assessed by the PsAID questionnaire in routine clinical practice.

## Methods

This was an observational, cross-sectional, multicenter study carried out at twenty-five rheumatology outpatient clinics over the whole of Spain. The study included outpatients of both genders over 18 years of age diagnosed with PsA according to the Classification for Psoriatic Arthritis (CASPAR) criteria [13] with at least 1 year of disease duration, with hand and foot radiological tests carried out during the 6 months prior to the study visit, and receiving treatment with biological and conventional synthetic (cs) disease-modifying anti-rheumatic drugs (DMARDs).

All patients provided their informed written consent. In accordance with Spanish recommendations, the study was approved by the Clinical Research Ethics Committee of La Fe Hospital and was conducted in accordance with the principles contained in the Declaration of Helsinki for studies in humans. Data were collected between May 2014 and February 2015 at a single visit.

Patient data collection included demographics and clinical characteristics (age, sex, body mass index, educational level and employment status, toxic habits, and comorbidities), detailed PsA clinical history (evolution time of PsA, time from onset of skin and articular symptoms, pattern of PsA at onset (peripheral, axial, mixed), enthesitis, dactylitis, involvement of distal interphalangeal joint, familial history (psoriasis, PsA, ankylosing spondylitis, others), C-reactive protein (CRP) levels, erythrocyte sedimentation rate (ESR), positive human leukocyte antigen-B27 (HLA-B27)), and record of current use of medications (nonsteroidal anti-inflammatory drugs (NSAIDs), infiltrations, biological and csDMARDs, and corticoids). Radiological findings (erosions in hands and feet, joint space narrowing on hands or feet, sacroilitis, syndesmophytes) from radiological tests carried out during the 6 months prior to the study visit were also recorded. The Psoriasis Area Severity index (PASI) [14] was assessed. In addition, patients completed self-reported questionnaires including the Health Assessment Questionnaire (HAQ) [15], the Bath Ankylosing Spondylitis Disease Activity Index (BASDAI) [16], and the PsAID [12].

Patients were considered in MDA when they met ≥5 of the following criteria: tender joint count ≤1, swollen joint count ≤1, PASI score ≤1 or body surface area ≤3%, patient pain visual analog scale (VAS) score ≤15, patient global disease activity VAS score ≤20, HAQ score ≤0.5, and tender entheseal points ≤1.

The PsAID questionnaire [12] reflects the impact of PsA from the perspective of the patient. It is comprised of 12 physical and psychological domains. Each domain is rated from 0 to 10 with a different weighting. The total score is divided by 20. The final score has a range from 0 (best status) to 10 (worst status) with a cutoff of 4 [12].

### Statistical methodology

A descriptive statistical analysis of all the variables was performed, including central tendency and dispersion measures for continuous variables, and absolute and relative frequencies for categorical variables. Patients were analyzed and distributed into two groups according to MDA status. Student's *t* test, Mann-Whitney *U* test or Kruskall Wallis *H* test were used to compare quantitative variables and Pearson’s chi-square or Fisher exact tests were used for qualitative variables. Univariate and multivariate models were carried out to identify factors independently associated with MDA. Tests were two-tailed with a significance level of 5%. Data were analyzed using SPSS V19.0 statistical software.

## Results

**Fig. 1:**
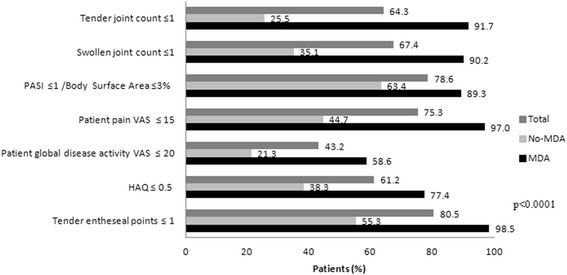
MDA criteria according to MDA state. *HAQ* Health Assessment Questionnaire, *MDA* minimal disease activity, *PASI* Psoriasis Area and Severity Index, *VAS* Visual analogue scale

Of the 227 included patients, 133 (58.6%) were in MDA at the study visit. Figure 1 shows the proportion of patients that fulfilled each MDA criterion according to MDA state. The most common active domains were global disease activity VAS score ≤20 and HAQ <0.5, achieved by 58.6% and 77.4% of MDA patients, respectively (Fig. 1).

Demographic and clinical characteristics of the study population with respect to the presence of MDA are shown in Table 1. Most of the patients were men (54.2%) with a mean (SD) age of 53.2 (12.4) years. Overall, 57.7% (MDA 47.9% vs. non-MDA 57.4%; *p* = 0.197) of patients suffered from at least one concomitant disease, the most frequent being dislipemia (MDA 33.1% vs. non-MDA 26.6%; *p* = 0.295), hypertension (MDA 26.3% vs. non-MDA 28.7%; *p* = 0.688), and obesity (MDA 24.1% vs. non-MDA 17.0%; *p* = 0.201; Table 1).

**Table 1:** Demographic and clinical characteristics of the study population

	Total *n* = 227	MDA *n* = 133	No MDA *n* = 94	*p*
Male, *n* (%)	123 (54.2)	82 (61.7)	41 (43.6)	<0.05
Age, mean (SD), years	53.2 (12.4)	53.5 (13.3)	52.8 (10.9)	NS
Smoker, *n* (%)	41 (18.1)	19 (14.3)	22 (23.4)	NS
Comorbidities, *n* (%)				
Dyslipemia	69 (30.4)	44 (33.1)	25 (26.6)	NS
HBP	62 (27.3)	35 (26.3)	27 (28.7)	NS
Obesity	48 (21.1)	32 (24.1)	16 (17.0)	NS
DM	23 (10.1)	15 (11.3)	8 (8.5)	NS
PsA characteristics				
PsA clinical pattern, *n* (%)				NS
Axial	8 (3.5)	3 (2.3)	5 (5.3)	
Peripheral	189 (83.3)	113 (85.0)	76 (80.9)	
Mixed	30 (13.2)	17 (12.8)	13 (13.8)	
Familial history, *n* (%)				
Psoriasis	112 (49.3)	64 (48.1)	48 (51.1)	NS
PsA	28 (12.3)	10 (7.5)	18 (19.1)	<0.05
Ankylosing spondylitis	2 (0.9)	2 (1.5)	0 (0.0)	NS
PsA duration, mean (SD), years	9.6 (7.7)	9.80 (8.1)	9.38 (7.3)	NS
Skin symptoms duration, mean (SD), years	22.1 (14.7)	20.6 (14.3)	24.2 (15.1)	NS
Articular symptoms duration, mean (SD), years	12.1 (9.3)	11.8 (8.8)	12.7 (9.9)	NS
PsA status at study visit				
Radiologic findings				
Erosions in hands, *n* (%)	83 (36.6)	41 (30.8)	42 (44.7)	<0.05
Joint in hands with erosion, mean (SD)	4.3 (4.2)	4.9 (4.5)	3.7 (3.9)	NS
Erosions in feet, *n* (%)	67 (29.5)	38 (28.6)	29 (30.9)	NS
Joint in feet with erosion, mean (SD)	3.7 (3.4)	3.9 (3.3)	3.5 (3.7)	NS
PsAID, mean (SD)	4.9 (4.5)	3.3 (3.1)	7.1 (5.2))	<0.001
PASI, mean (SD)	1.6 (3.8)	0.9 (1.6)	2.8 (5.7)	<0.05
BASDAI*, mean (SD)	2.8 (2.4)	2.0 (1.8)	3.6 (2.5)	<0.001
HAQ, mean (SD)	0.8 (0.6)	0.3 (0.5)	0.5 (0.6)	<0.001

*BASDAI* Bath Ankylosing Spondylitis Disease Activity Index, *DM* diabetes mellitus, *HAQ* Health Assessment Questionnaire, *HBP* high blood pressure, *MDA* minimal disease activity, *NS* not significant, *PASI* Psoriasis Area and Severity Index, *PsA* psoriatic arthritis, *PsAID* Psoriatic Arthritis Impact of Disease, *SD* standard deviation. ﻿*Performed only in subjects with axial disease

Of the total patients, 54.6% were employed (MDA 60.9% vs. non-MDA 45.7%) and 5.7% had temporary or full disability to work due to PsA (MDA 1.5% vs. non-MDA 11.7%; *p* < 0.001). Regarding PsA condition, 83.3% of the patients were diagnosed with peripheral disease (oligoarticular 51.6%, polyarticular 48.4%). Dactylitis, enthesitis, and distal interphalangeal joints were detected in 49.3%, 35.7%, and 41.0% of patients, respectively.

Overall, 49.3% of the patients had familial history of psoriasis, and 12.3% had a history of PsA, which was significantly more frequent in non-MDA patients than in those who achieved the MDA state (19.1% vs. 7.5%; *p* < 0.05; Table 1). Disease duration was 9.6 (7.7) years and the musculoskeletal symptoms onset occurred nearly 10 years after the skin symptoms appeared (Table 1). At the study visit, MDA patients had fewer hand erosions than non-MDA patients (30.8% vs. 44.7%; *p* < 0.05; Table 1). The mean (SD) CRP level was 3.6 (6.2) mg/L, (MDA 2.8 (3.9) mg/L vs. non-MDA 4.7 (8.2) mg/L; *p* < 0.05), the mean (SD) ESR value was 14.76 (12.4) mm/h, and 23 (17.6%) of 131 patients tested for HLA-B27 were positive.

**Fig. 2:**
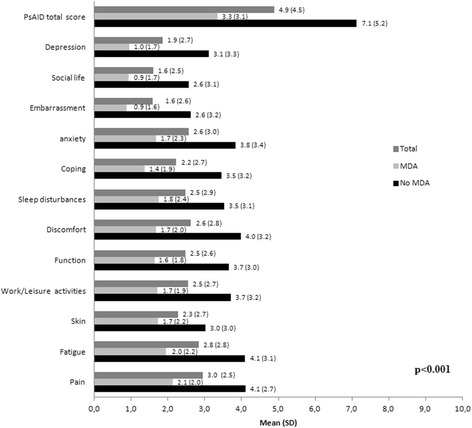
Psoriatic Arthritis Impact of Disease (*PsAID*) score according to minimal disease activity (*MDA*) state

MDA patients had a significantly lower impact of the disease as measured by PsAID (MDA 3.3 (3.1) vs. non-MDA 7.1 (5.2); *p* < 0.001; Table 1). Eighty-eight (66.7%) of MDA patients vs. 34 (37.4%) of non-MDA patients obtained a PsAID score <4 (*p* < 0.0001). Statistically significant differences were observed between MDA and non-MDA patients for all PsAID domains (*p* < 0.001; Fig. 2). Similarly, patients with MDA presented a significantly lower total score on PASI, BASDAI, and HAQ questionnaires compared with those who had not achieved MDA (*p* < 0.001; Table 1).

Multivariate analysis showed that male gender increased the odds of having achieved MDA (odds ratio (95% confidence interval) 2.75 (1.47–5.12); *p* = 0.001) as well as living a sedentary life (3.13 (1.50–6.53); *p* = 0.002), while having a familial history of PsA (0.39 (0.16–0.94); *p* = 0.038), current elevated CRP level (0.92 (0.86–0.98); *p* = 0.010) or use of corticosteroids (0.33 (0.15–0.74); *p* = 0.007) decreased the odds of having achieved MDA (Table 2).

**Table 2:** Multivariate logistic regression analysis

	*p* value	Adjusted OR (95% CI)
Sex (men)	0.001	2.746 (1.473–5.119)
Sedentary lifestyle	0.002	3.127 (1.497–6.534)
PsA familial history	0.038	0.387 (0.159–0.938)
C-reactive protein	0.010	0.920 (0.864–0.980)
Corticoids	0.007	0.335 (0.151–0.745)

*CI* confidence interval, *PsA* psoriatic arthritis, *OR* odds ratio

Treatments at the study visit are shown in Table 3. MDA was achieved by 62.7% of the patients who received anti-TNF alone, by 52.5% receiving anti-TNF plus csDMARDs treatment, and by 62.7% receiving monotherapy with csDMARDS (Table 3). Overall, the most frequent anti-TNFs used were etanercept (37.5%) and adalimumab (31.3%), and among the csDMARDs the most common was methotrexate (76.0%).

**Table 3:** PsA treatment at study visit

	Total	MDA	No MDA	*p*
Treatment pattern, *n* (%)				NS
Anti-TNF monotherapy	110	69 (62.7)	41 (37.3)	
DMARDS and anti-TNF	61	32 (52.5)	29 (47.5)	
DMARDS monotherapy	51	32 (62.7)	19 (37.3)	
Anti-TNF*, *n* (%)	112	64 (57.1)	48 (42.9)	NS
Etanercept	42	26 (61.9)	16 (38.1)	
Adalimumab	35	22 (62.9)	13 (37.1)	
Infliximab	15	6 (40.0)	9 (60.0)	
Golimumab	13	7 (53.8)	6 (46.2)	
Mean (SD) time, months	45.7 (34.7)	50.5 (35.0)	40.2 (33.8)	NS
DMARDS*, *n* (%)	171	101 (59.1)	70 (40.9)	NS
Metotrexate	130	76 (58.5)	54 (41.5)	
Leflunomide	32	17 (53.1)	15 (46.9)	
Mean (SD) time, months	73.7 (70.4)	71.8 (71.8)	76.4 (69.7)	NS
NSAIDs**, *n* (%)	114	62 (54.4)	52 (45.6)	NS
Infiltrations**, *n* (%)	48	27 (56.3)	21 (43.8)	NS
Corticoids**, *n* (%)	39	16 (41.0)	23 (59.0)	<0.05

*Monotherapy or combination therapy

**Combination therapy

*anti-TNF* anti-tumor necrosis factor, *DMARDS* disease-modifying anti-rheumatic drugs, *NS* not significant, *NSAIDs* nonsteroidal anti-inflammatory drugs

Statistically significant differences were not observed between non-MDA and MDA patients regarding use of any kind of treatment with the exception of corticoids (Table 3). The proportion of patients who received corticoids was significantly higher among non-MDA than MDA patients (59% non-MDA vs. 41% MDA; *p* < 0.05; Table 3).

## Discussion

Remission or low disease activity is the goal of therapy in PsA. In this study, 58.6% of the patients were in a MDA state. This prevalence is in concordance with that reported in other studies [6–8]. Recent prospective studies, also carried out in the clinical setting, demonstrated that up to 64% of patients treated with anti-TNFα drugs achieved MDA after 12 months [6–8]. A similar rate (60%) was also reported in a PsA cohort of 344 patients receiving biological and non-biological DMARDs [17].

The less frequently achieved MDA criteria by the MDA population were patient global disease activity VAS score ≤20 and HAQ <0.5; this latter measure focused on physical disability and pain. This finding is in line with previous results [6], and might reflect the patient’s perception not only of damage but also of active disease. In fact, meeting MDA criteria does not exclude the presence of a small amount of disease activity [17]. However, MDA is a good therapeutic target in PsA, as patients who reach this state have better functional status and lower impact of disease.

In line with our results, male sex has been identified previously as a predictor not only of MDA [18] but also of response to treatment with anti-TNFα therapy [6–8]. However, we found that a sedentary lifestyle is a factor associated with MDA, which could be in contradiction with previous studies where obesity and metabolic syndrome have been associated with a lower probability of achieving MDA [19–21]. Indeed, the benefit of programs that encourage physical activity has been reported recently [22]. Although unexpected and controversial, according to the hypothesis that mechanical stress is associated with musculoskeletal inflammation, a sedentary lifestyle in PsA could reduce clinical complications associated with strenuous physical activity [23].

There has been evidence of genetic predisposition to PsA, although there is no specific gene for the disease [24, 25]. In our study, the presence of a familial history of PsA was related with non-MDA. It may represent an effect of genetics both in the severity and in the capability to respond to drugs, although it is an open thesis that calls for additional research. On the contrary, in our study having a familial history of psoriasis did not present a relationship with MDA. Similarly, a recent study showed that the severity of psoriasis and the proportion of patients with comorbidities were not affected by the presence of a family history of psoriasis [26].

In PsA, the most common inflammatory indicators (ESR and CRP) used for the evaluation of disease activity in rheumatoid arthritis are within normal levels in half of the patients [27]. However, when these two markers are increased, their utility is undeniable [27]. Baseline high CRP and ESR are predictors for MDA [6]. Conversely, in this cross-sectional study, we found that having increased CRP level at the study visit has a very slight relationship to non-MDA status. Being a transversal study, it may only reflect the patients who are in a state of greater activity.

We also identified use of therapy with corticoids as a possible factor related to non-MDA state. This could reflect that physicians were more likely to prescribe corticosteroids in severe/difficult cases. On the other hand, several authors have indicated that systemic therapy with corticoids hinders the control of skin manifestations and should therefore not be used routinely, but only for a limited period of time [28, 29]. Systemic use of corticosteroids improves skin psoriasis, but their withdrawal triggers relapses in the form of a “rebound effect”, producing recurrence of the skin manifestations or transformation into the generalized pustular form [28, 29].

Heterogeneity and complexity of PsA are challenging for the clinical measure of the disease, both from the patient’s perspective and from the physician’s view. Patients with PsA experience functional impairment and reduced quality of life, so patient global assessment in PsA is explained mainly by the physical, but also by the psychological aspect, of the disease [30]. PROs, such as the novel PsAID questionnaire [12], are important instruments to evaluate healthcare interventions and to reflect the impact of PsA on patients’ lives. There are few studies that have reported results with this tool [31]. In the present study, we observed that MDA state and lower impact of the disease are associated. Thus, statistically significant differences were observed between MDA and non-MDA patients in all physical and psychological PsAID domains. These findings suggest that PsAID information seems to be in consonance with the MDA state and could be a useful tool for assessing PsA from the patient’s point of view.

An adequate therapeutic approach for PsA depends on the type and severity of the skin and joint involvements [32]. Anti-TNF drugs have been shown to significantly inhibit joint damage [33–35]. In the present study, MDA was achieved by a significant proportion of patients (63%) receiving anti-TNF or csDMARD monotherapy. Interestingly, we found a lower, but not meaningful, proportion of patients receiving anti-TNF concomitantly with csDMARDS (52.5%) in the MDA state compared with those treated with anti-TNF alone. This could be due to concomitant therapy being administered to more severe patients who have more difficulty achieving a MDA state.

Moreover, ad hoc analysis results from clinical trials indicated that concomitant use of methotrexate and anti-TNF was not more effective in achieving a response [32, 36]. A recent study showed that methotrexate administered in combination with etanercept in patients with PsA may not provide significantly greater improvement in arthritis or psoriasis symptoms than etanercept monotherapy [36]. Despite the advances in therapy for PsA, there remain many patients who fail to respond and lose efficacy over time [32]. In the present study, 41.4% of the patients did not achieve the MDA state, although statistically significant differences were not observed between non-MDA and MDA patients regarding the use of non-biological DMARDs and biological DMARDs. Some authors suggested that the TNFα pathway might not have a pivotal role in driving the inflammation in such patients, and these patients might benefit from other treatments targeted at different inflammatory pathways that are implicated in the disease activity [7, 32].

Some limitations derived from the cross-sectional nature of this study should be borne in mind. PsA is known to be a heterogeneous disorder, with progression highly variable between individuals. The data presented here are a photograph of a specific moment of the state of illness of patients with a long evolution of the disease. We cannot be sure what proportion of patients achieved MDA state due to the natural history of the disease and what success treatment had on outcome. Thus, it is difficult to know whether the management of these patients was as appropriate as it should have been. Furthermore, we do not know the proportion of patients who reached sustained MDA and how long they remained in this state. A further limitation is related to the study being designed to include patients on conventional or biological DMARDs, representing the majority of PsA cases followed in the Rheumatology Department. PsA patients that only require NSAIDs or corticosteroids are usually followed in general practice and therefore they were not included.

Despite these limitations, this multicenter study shows the treatment response of a representative Spanish population with PsA in a routine clinical setting. In addition, a further strength of this study is the use of the novel PsAID questionnaire, which allowed a precise interpretation of the patient-perceived impact of the disease.

To summarize, in this study we report that a significant proportion of PsA patients achieve a MDA state in normal clinical practice, reflecting a management of PsA in Spain similar to that which has been published in other countries. The PsAID could be an additional useful tool to assess PsA interventions in clinical practice, although further studies are needed to confirm it.

## Conclusions

This study supports the use of MDA and PsAID as two of the main assessment tools for PsA in routine clinical practice.

## Abbreviations

BASDAI: Bath Ankylosing Spondylitis Disease Activity Index
CASPAR: Classification for Psoriatic Arthritis
CRP: C-reactive protein
csDMARD: Conventional synthetic disease-modifying anti-rheumatic drug
DMARD: Disease-modifying anti-rheumatic drug
ESR: Erythrocyte sedimentation rate
HAQ: Health Assessment Questionnaire
HLA-B27: Human leukocyte antigen-B27
MDA: Minimal disease activity
NSAID: Nonsteroidal anti-inflammatory drug
PASI: Psoriasis Area Severity Index
PRO: Patient-reported outcome
PsA: Psoriatic arthritis
PsAID: Psoriatic Arthritis Impact of Disease
TNF: Tumor necrosis factor
VAS: Visual analog scale
